# Predicting the likelihood of carrying a *BRCA1* or *BRCA2* mutation in Asian breast cancer patients

**DOI:** 10.1200/JCO.21.01647

**Published:** 2022-02-10

**Authors:** Boon Hong Ang, Weang Kee Ho, Eldarina Wijaya, Pui Yoke Kwan, Pei Sze Ng, Sook Yee Yoon, Siti Norhidayu Hasan, Joanna M. C. Lim, Tiara Hassan, Mei-Chee Tai, Jamie Allen, Andrew Lee, Nur Aishah Mohd Taib, Cheng Har Yip, Mikael Hartman, Swee Ho Lim, Ern Yu Tan, Benita K. T. Tan, Su-Ming Tan, Veronique K. M. Tan, Peh Joo Ho, Alexis J. Khng, Alison M. Dunning, Jingmei Li, Douglas F. Easton, Antonis C. Antoniou, Soo Hwang Teo, #Malaysian Breast Cancer Genetic Study (MyBrCa) and Singapore Breast Cancer Cohort (SGBCC) contributors are listed in the Acknowledgments section.

**Affiliations:** 1 Cancer Research Malaysia, Subang Jaya, Malaysia; 2 Faculty of Science and Engineering, School of Mathematical Sciences, University of Nottingham Malaysia, Jalan Broga, Semenyih, Selangor, Malaysia; 3 Department of Public Health and Primary Care, Centre for Cancer Genetic Epidemiology, University of Cambridge, Cambridge, United Kingdom; 4 Faculty of Medicine, University Malaya Cancer Research Institute, University of Malaya, Jalan Universiti, Kuala Lumpur, Malaysia; 5 Department of Surgery, Faculty of Medicine, University of Malaya, Jalan Universiti, Kuala Lumpur, Malaysia; 6 Subang Jaya Medical Centre, Subang Jaya, Malaysia; 7 Department of Surgery, National University Hospital and NUHS, Singapore, Singapore; 8 Breast Department, KK Women’s and Children’s Hospital, Singapore, Singapore; 9 Department of General Surgery, Tan Tock Seng Hospital, Singapore, Singapore; 10 Lee Kong Chian School of Medicine, Nanyang Technological University, Singapore, Singapore; 11 Institute of Molecular and Cell Biology, Singapore, Singapore; 12 Division of Surgery and Surgical Oncology, National Cancer Centre Singapore, Singapore, Singapore; 13 Department of Breast Surgery, Singapore General Hospital, Singapore, Singapore; 14 Department of General Surgery, Sengkang General Hospital, Singapore, Singapore; 15 Division of Breast Surgery, Department of General Surgery, Changi General Hospital, Singapore, Singapore; 16 Laboratory of Women’s Health and Genetics, Genome Institute of Singapore, Singapore, Singapore; 17 Department of Oncology, Centre for Cancer Genetic Epidemiology, University of Cambridge, Cambridge, United Kingdom

## Abstract

**Purpose:**

With the development of PARP inhibitors for treatment of cancer patients with an altered *BRCA1* or *BRCA2* gene, there is an urgent need to ensure that there are appropriate strategies for identifying mutation carriers whilst balancing the increased demand for and cost of cancer genetics services. To date, the majority of mutation prediction tools have been developed in women of European descent where the age and cancer-subtype distributions are different from that in Asian women.

**Methods:**

In this study, we built a new model (ARiCa: **A**sian **Ri**sk **Ca**lculator) for estimating the likelihood of carrying a pathogenic variant in *BRCA1* or *BRCA2* gene, using germline *BRCA* genetic testing results in a cross-sectional population-based study of 8,162 Asian breast cancer patients. We compared the model performance to existing mutation prediction models. The models were evaluated for discrimination and calibration.

**Results:**

ARiCa included age of diagnosis, ethnicity, bilateral breast cancer, tumour biomarkers, and family history of breast cancer or ovarian cancer as predictors. The inclusion of tumour grade improved significantly the model performance. The full model was calibrated (Hosmer-Lemeshow p-value=0.614) and discriminated well between *BRCA* and non-*BRCA* pathogenic variant carriers (Area Under Receiver Operating Curve 0.80, 95% Confidence Interval=0.75-0.84). Addition of grade to the existing clinical genetic testing criteria targeting breast cancer patients below 45 years reduced the proportion of patients referred for genetic counselling and testing from 37% to 33% (p-value=0.003), thereby improving the overall efficacy.

**Conclusion:**

Population-specific customisation of mutation prediction models and clinical genetic testing criteria improved the accuracy of BRCA mutation prediction in Asian patients.

## Introduction

Germline genetic testing for *BRCA1* and *BRCA2* (*BRCA*) has enabled risk management for individuals at elevated cancer risk, and with the advent of PARP inhibitor treatment, enabled treatment selection with improved outcomes.^1^ In high resource countries, clinical genetics services are well established and patients are referred for germline *BRCA* genetic testing using criteria based on age of onset of cancer, breast cancer histology, and cancer family history.^2,3^

Similar genetic testing criteria have been incorporated into clinical practice guidelines in Asian countries, but these pose significant resource challenges, particularly in low- and middle-income Asian countries with limited clinical genetics services.^4^ Notably, because of the shifting reproductive and behavioural patterns, the incidence in many Asian countries have doubled or tripled in the past 40 years.^5^ This dramatic increase in incidence in younger generations means that the mean age of diagnosis for breast cancer in Asian women is approximately 10 years younger than that in European women,^6,7^ thus, a higher proportion of breast cancer patients fulfil clinical genetic testing criteria for referral, exacerbating the challenges in access to genetic services in Asian countries.^4^

In European or North American populations, models have been developed for predicting the likelihood of carrying germline *BRCA* pathogenic variants (PVs), mostly using data of breast cancer patients ascertained through genetic clinics, and these models are well-calibrated for these populations.^8,9,10,11,12,13,14,15^ Evaluation of such models in high-risk breast cancer patients of Asian descent living in North America^16^ or in Asia^17,18,19,20,21^ showed that these models underestimated the proportion of *BRCA* PVs carriers, especially for *BRCA2* PVs carriers^16,17,20,21^ and for breast cancer patients with no family history of breast cancer.^17,18^ Recently, a BRCA carrier prediction algorithm (KOHCal) was developed in South Korea, based on high-risk breast cancer patients and was found to have better discrimination and calibration in South Koreans than models built on women of European descent.^22^

With the approval of PARP inhibitors for treatment of breast and ovarian cancer patients with *BRCA* PVs, there is an urgent need to determine the performance of these models in diverse populations. To date, no studies have evaluated the performance of clinical genetic testing criteria or developed a BRCA carrier prediction model in a population-based study of Asian breast cancer patients. In this study, we evaluated the performance of existing BRCA carrier prediction models, developed a new prediction model, and customised clinical genetic testing criteria in a population-based study of 8,162 Asian breast cancer patients from Malaysia and Singapore unselected for age of diagnosis and family history of cancer.

## Methods

### Study population

The study participants were women diagnosed clinically with breast cancer (invasive and non-invasive) who were recruited in the Malaysian Breast Cancer Genetic (MyBrCa) study^23^ and the Singapore Breast Cancer Cohort (SGBCC) study. Cases were recruited from two hospitals in Malaysia and six hospitals in Singapore. Germline DNA were sequenced in two batches, using targeted sequencing panels described previously.^24^ Carriers of pathogenic variants in non-BRCA genes were treated as non-carriers.

### Statistical analyses

#### Existing BRCA carrier prediction models

Three existing BRCA carrier prediction models were evaluated in this study; two empirical models (PENNII, KOHCal) and a genetic risk model BOADICEA 5.0.^25^ Model performance was determined based on model calibration, assessed using Hosmer-Lemeshow (HL) test, and discrimination, assessed using area under receiver operating curve (AUC).

#### Development and validation of population-specific BRCA carrier prediction model

The study sample was randomly split into training and validation sets, comprising 70% and 30% of the samples, respectively. Candidate predictors of BRCA PV included age of breast cancer diagnosis, ethnicity, bilateral breast cancer, pathological features, and family history of breast or ovarian cancer (Supplementary Table 1). Missing data in the training set were imputed using multiple imputation by chained equations, whilst missing data in the validation set were imputed using single or multiple imputation by chained equations, under the missing at random (MAR) assumption.^26,27^ Given that multiple imputation generates more than one imputed dataset, results for single imputation in the validation dataset is presented in the main Figures and Tables, with results of multiple imputation included in Supplementary data where relevant. Additional sensitivity test was performed to ensure that the validation test results are comparable after single and multiple imputations. BRCA carrier prediction models were built based on logistic regression method using the training set. Model calibration and discrimination were evaluated in the validation set using HL test and AUC, respectively. The optimal carrier probability threshold for genetic testing was chosen based on the intersection of sensitivity and specificity curves.^28^

#### Customisation and evaluation of existing clinical criteria for germline BRCA genetic testing

Modified Clinical Criteria (MCC) were developed starting with the MCGplus Criteria^2^ by considering combinations of age of diagnosis of proband in 5 year intervals, with and without considering grade, resulting in a total of 96 different categories. The efficacy of MCC was evaluated in the validation set based on detection ratio (number of patients to be screened to detect one carrier).

All the data were analysed using Stata version 13.0 (Stata Corp., College Station., Texas, USA) and a p-value < 0.05 (two-tailed) was deemed to be statistically significant. See Supplemental Methods for more details.

## Results

### Study population characteristics

In this cross-sectional population-based study of 8,162 breast cancer patients, 323 (4.0%) had germline *BRCA1* or *BRCA2* PVs (Supplementary Table 1). The majority of patients were Chinese (75.4%), with a mean age of diagnosis of 52.3 years (SD=10.77). Compared to Chinese women, Indian women had a higher proportion of HR- and TNBC breast cancer cases, whereas Malay women had higher proportions of HER2+ and Luminal B breast cancer cases. There was a higher proportion of carriers amongst Indian and Malay women. Whilst the tumour characteristics tested were significantly associated with *BRCA1* status, these were also associated with BRCA2 status, with the exception of ER, PR, HR, and TNBC (Supplementary Table 2).

### Development and validation of population-specific BRCA carrier prediction model

Prediction models were developed using 5,714 breast cancer cases (228 *BRCA* carriers) and validated using 2,448 cases (95 *BRCA* carriers) (Supplementary Fig 1). Collinearity tests showed that ER, PR, HER2, TNBC, HR, and immune-histochemical subtypes were correlated (correlation coefficients, r>0.40). Hence, six combinations of tumour biomarkers along with the remaining predictors were considered in the analyses: (a) TNBC, (b) ER, (c) ER and HER2, (d) HR and HER2, (e) HER2, and (f) immune-histochemical subtypes. The best-performing model was selected based on the highest AUC and the lowest non-significant HL score in the validation set (Supplementary Table 3). Model (a) (AUC=0.86, HL=2.63) and Model (e) (AUC=0.75, HL=10.89) were the best-performing models for *BRCA1* and *BRCA2* PVs carrier status, whereas Model (c) was the best-performing model for overall *BRCA* (AUC=0.80, HL=5.43). The predictive performance of Model (c) by mutation-type were similar to the respective best-performing models (*BRCA1*: Model (c) versus Model (a) – AUC (HL): 0.86 (4.33) versus 0.86 (2.63); *BRCA2:* Model (c) versus Model (e) – AUC (HL): 0.75 (12.15) versus 0.75 (10.89)). Analyses after multiple imputation showed similar results. Hence, Model (c), subsequently referred to as ARiCa (**A**sian **Ri**sk **Ca**lculator), was selected as the final model for predicting overall *BRCA* PVs carrier status. We evaluated the performance of ARiCa by ethnicity and found that the model had high discriminatory power and well-calibrated across ethnic groups (Supplementary Table 4).

In ARiCa, younger age of diagnosis, Indian ethnicity, bilateral breast cancer, ER-negativity, HER2-negativity, higher grade, and presence of first degree family history of breast or ovarian cancer were associated with overall *BRCA* PVs carrier status (Table 1). These variables were also associated with *BRCA1* PVs carrier status except grade, whereas *BRCA2* was only associated with younger age, HER2-negativity, higher grade and first degree family history of breast cancer. Notably, both Malay and Indian ethnicities were associated with higher odds of being *BRCA1* PVs carriers compared to Chinese ethnicity.

We determined the optimal carrier probability threshold for ARiCa in the validation set as the intercept of sensitivity and specificity (Supplementary Fig 2). At the optimal threshold, corresponding to a mutation prevalence of 4%, 31% (95%CI=29-33) of breast cancer patients would require germline *BRCA* genetic testing and 71% (95%CI=61-80) of *BRCA* PVs carriers would be identified (Supplementary Table 5). Performance of ARiCa were consistent across imputed validation sets after multiple imputation.

### Comparison of BRCA carrier prediction models

**Fig. 1:**
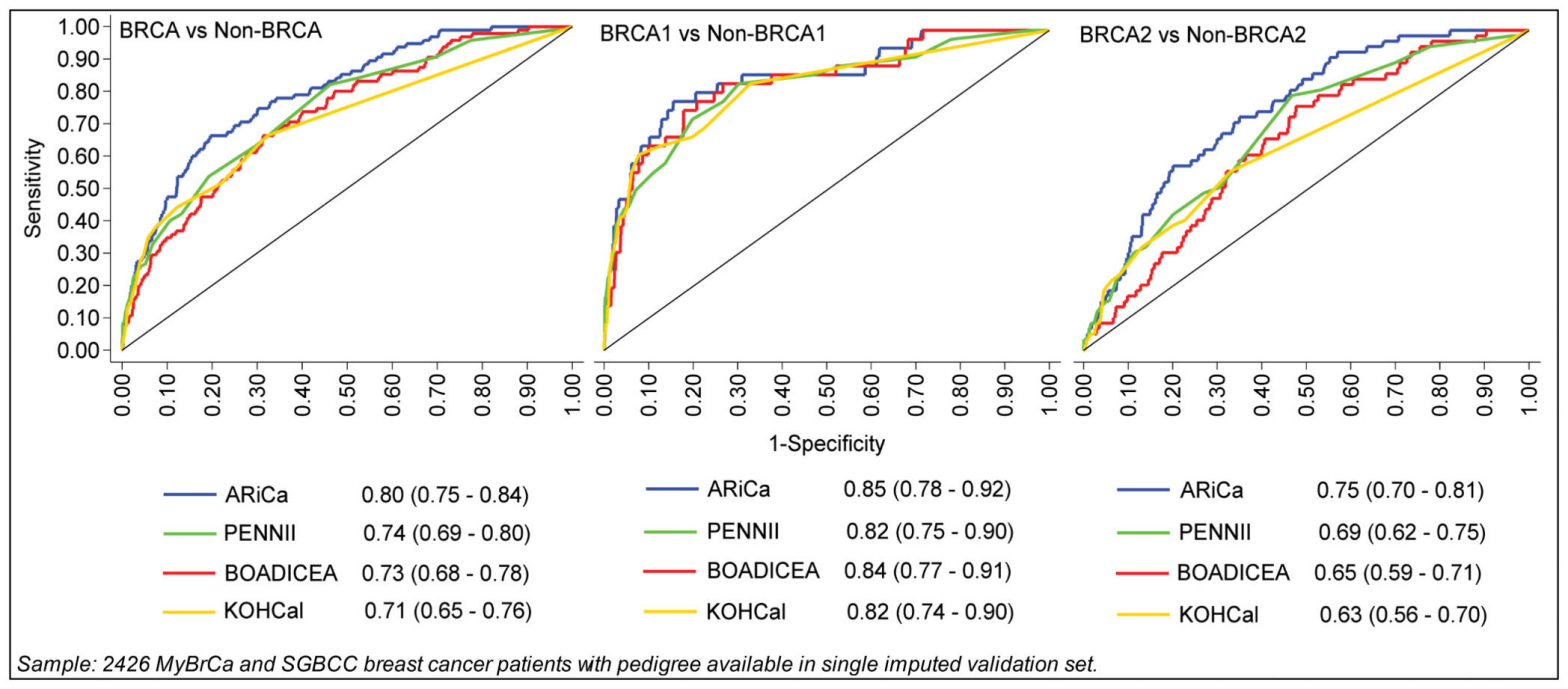
ROC curves of BRCA carrier prediction models Sample: 2426 MyBrCa and SGBCC breast cancer patients with pedigree available in single imputed validation set.

We compared the performance of ARiCa with models which have been developed in other populations using data for 2,426 patients from the validation set for whom data are available for variables required in all considered models. For overall *BRCA*, ARiCa had the highest AUC (0.80), followed by PENNII (0.74), BOADICEA (0.73), and KOHCal (0.71) (Fig 1). The AUCs for *BRCA1* were similar across models, but ARiCa had significantly better discriminatory ability than PENNII, BOADICEA, and KOHCal for *BRCA2* (0.75, 0.69, 0.65, and 0.63 respectively).

**Fig. 2:**
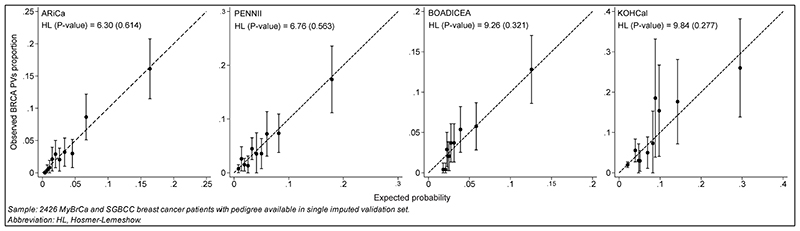
Observed proportion and expected probability of BRCA carrier prediction models Sample: 2426 MyBrCa and SGBCC breast cancer patients with pedigree available in single imputed validation set. Abbreviation: HL, Hosmer-Lemeshow.

All models were well-calibrated; ARiCa had the lowest HL for overall *BRCA* (Fig 2). There was no significant difference between the observed proportion and expected probability for *BRCA1* and *BRCA2* PVs carriers and a majority were distributed close to the bisector (Supplementary Fig 3).

We compared the efficacy measures (sensitivity, specificity) at the optimal and the conventional 10% and 20% thresholds.^17,21,29^ All models had poor sensitivity at the 10% and 20%, so we focused on the lower optimal thresholds (Table 2).^17^ At the respective optimal thresholds for each model, the sensitivities of all models for overall *BRCA* were 63-71% and the specificities were 67-71%. Whilst all models achieved a sensitivity of 83% for *BRCA1*, KOHCal (56%), BOADICEA (56%), and PENNII (51%) had lower sensitivity for *BRCA2* than ARiCa (66%). ARiCa achieved relatively high sensitivity and specificity for overall *BRCA* (71%, 71%), *BRCA1* (83%, 70%), and *BRCA2* (66%, 70%) at the optimal threshold.

### Customisation and evaluation of existing clinical criteria for germline *BRCA* genetic testing

We evaluated the NCCN and Mainstreaming Cancer Genetics (UK) clinical genetic testing criteria in the validation set. Applying the NCCN and MCG Criteria would lead to 37% and 39% being referred with 72% and 69% of *BRCA* PVs carriers identified, respectively. Addition of family history variables to the MCG Criteria (MCGPlus) increased the screening rate from 39% to 49% and improved the detection rate from 69% to 81% (data not shown). Whilst the expanded NCCN Criteria detected the highest detection rate (96%), more than three-quarters of breast cancer patients (88%) would need to be screened.

**Fig. 3:**
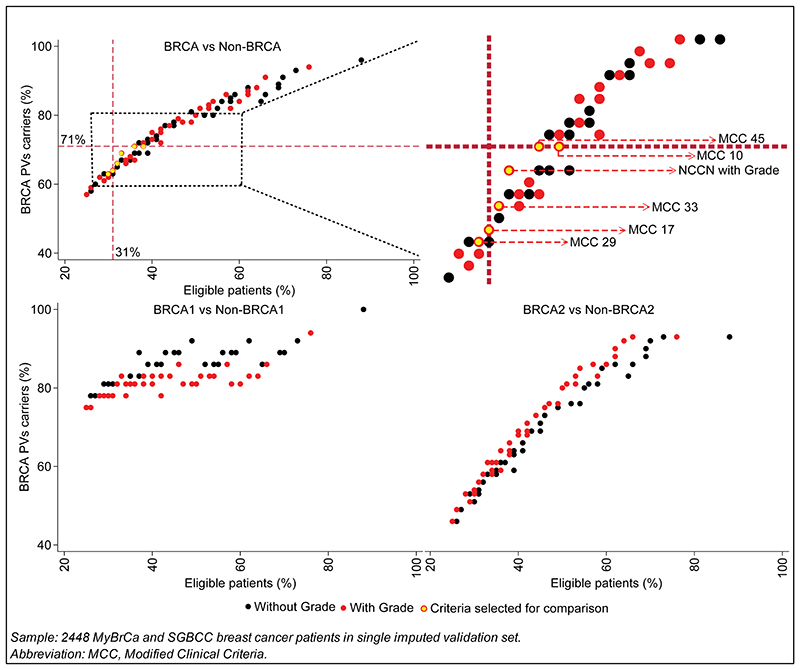
Eligible patients and BRCA PVs carriers detected for clinical criteria with and without grade Sample: 2448 MyBrCa and SGBCC breast cancer patients in single imputed validation set. Abbreviation: MCC, Modified Clinical Criteria.

Given that patients in this study had a younger age of diagnosis for breast cancer than those in the Western populations and grade was a significant predictor of *BRCA* PVs carrier status, we customised MCGplus Criteria by considering several combinations of age of diagnosis for breast cancer and higher-grade breast cancer (grade 2 or 3) to improve the overall efficacy. Applying 96 Modified Clinical Criteria (48 MCC with grade and 48 MCC without grade) (Fig 3), we found that the detection rates for *BRCA1* (78-92%) were higher than for *BRCA2* (46-93%). Notably, at similar detection rate, the addition of grade resulted in reduction in screening rate of 1% to 10% (average=4%) for overall *BRCA*. Similarly, the addition of grade resulted in reduction in screening rate of 3% to 12% (average=5%) for existing clinical genetic testing criteria. There was no difference in reduction rates between *BRCA1* and *BRCA2*.

We identified 3 clinical criteria categories (MCC 17, 29, 33) from Fig 3 with similar screening rates to ARiCa for overall *BRCA* (screening rate=31%) (Supplementary Table 5). These criteria had identical criteria for grade 2 or 3 breast cancer (≤40) and bilateral breast cancer (≤60), but they had different thresholds for age of diagnosis of proband with TNBC and family history of breast or ovarian cancer (Table 3).

We also identified 3 clinical criteria categories (NCCN with grade, MCC 10, 45) from Fig 3 with similar detection rates to ARiCa for overall *BRCA* (detection rate=71%) (Supplementary Table 5). These criteria had different combinations of age of diagnosis of proband with grade 2 or 3 breast cancer, TNBC, bilateral breast cancer, and family history of breast or ovarian cancer (Table 3).

All 6 modified criteria resulted in lower detection ratios, when compared to existing clinical genetic testing criteria (Expanded NCCN=24:1; MCGplus=15:1; MCG=14:1; NCCN=13:1) (Table 3). NCCN with grade outperformed the 5 modified criteria by achieving a higher detection rate (69%) at the lowest detection ratio (12:1). Nonetheless, all 6 modified criteria still underperformed ARiCa. For instance, MCC (17, 29, 33) had lower detection rates of 63-66% compared to 71% for ARiCa. Similarly, NCCN with grade and MCC (10, 45) had higher screening rate of 33-38% compared to 31% for ARiCa (Table 3).

## Discussion

Whilst germline *BRCA1* or *BRCA2* PVs testing has an established role in risk management, this is increasingly relevant in the selection of therapy.^1^ We showed that logistic regression models built based on a large Asian population-based study of breast cancer patients, unselected for age of diagnosis and family history, outperformed the genetic risk model (BOADICEA) developed using data on European-ancestry populations and the empirical models (PENNII, KOHCal) developed using breast cancer patients with early onset or familial breast cancer. The Modified Clinical Criteria (MCC) customised to the Asian breast cancer patients in combination with presence of grade were more efficient than existing clinical genetic testing criteria.

In multivariable regression analyses, we found that the risk factors significantly associated with *BRCA* PVs carrier status in this study were consistent with previously published findings from Asian countries, including younger age of diagnosis, bilateral breast cancer, ER-negative status, HER2-negative status, higher grade, and presence of first degree family history of breast cancer or ovarian cancer.^20,22,30,31,32,33,34,35,36,37,38^

We found that all the BRCA carrier prediction models and the Modified Clinical Criteria (MCC) were more sensitive (sensitivity/detection rate) and accurate (discrimination) for *BRCA1* than *BRCA2*, which is likely to be driven by the stronger association between *BRCA1* and the ER-negative status, TNBC subtype, and ovarian cancer family history.^39,40,41,42,43^ Indeed, several studies have previously demonstrated that the use of pathologic characteristics, namely ER and TNBC improved the sensitivity and discrimination for *BRCA1* when selecting individuals for germline genetic testing in high-risk breast cancer patients.^44,45,46,47,48^

All three existing models tested performed similarly in our study population (AUC=0.71-0.74) as previously reported in other Asian populations (AUC=0.69-0.76), but the AUCs were lower than those reported in women of European descent, especially for BOADICEA (AUC=0.77).^13,17,22,29^ BOADICEA had lower discriminatory ability for overall *BRCA* and *BRCA2*, consistent with the observation that BOADICEA performed better at lower thresholds because it underestimated carrier probability of Asian breast cancer patients with the lowest sensitivity at conventional thresholds, particularly those with germline *BRCA2* PVs.^17^ Nevertheless, BOADICEA outperformed PENNII for *BRCA1*.^17^ These observations are not surprising. Given that BOADICEA is a genetic risk model, it relies on population-specific parameters for breast cancer incidences, PV frequencies, and tumour-pathology distributions as input parameters. Customisation of BOADICEA using population-specific parameters and addition of tumour grade (a clear predictor of carrier status in our analysis) could substantially improve its discrimination.

In terms of calibration, models built on Asian populations had better calibration for *BRCA2*, whilst models built on women of European descent had better calibration for *BRCA1*. Notably, this was evident in KOHCal and BOADICEA that appeared to be calibrated with the lowest HL for *BRCA2* and *BRCA1*, respectively. A possible explanation could be due to the variation in mutation prevalence. Whilst *BRCA2* mutations are more common than *BRCA1* mutations in Asian, it is the opposite in many European populations.^17^

Notwithstanding BRCA carrier prediction models have good discrimination, there are challenges in their implementation in resource constrained settings. Clinical genetic testing criteria are likely to continue as a mainstay for referral of patients for genetic counselling and testing. In our evaluation, NCCN with grade had a sensitivity of 69% at a screening rate of 33%. This is marginally better than the existing NCCN and MCG criteria where at similar detection rates of 69-72%, about 37-39% patients would be referred for genetic counselling and testing. It is possible that this improvement is because of the age threshold for TNBC (≤60 vs no age restriction), bilateral breast cancer (46-50 vs ≤60), and first degree family history of breast cancer (46-50 vs no age restriction), but the inclusion of grade is also an important consideration. Indeed, higher grade was identified as a strong predictor not only for *BRCA1* PVs carriers but also for *BRCA2* PVs carriers.^42^ Previous studies have shown that inclusion of grade can improve the sensitivity and discrimination of germline *BRCA* PVs prediction in high-risk breast cancer patients.^44,45^ Given that *BRCA1* (91%) and *BRCA2* (89%) PVs carriers were of higher grade than in non-carriers (76%), future improvements in BRCA carrier prediction tools could include grade.^45,49^

### Limitations and strengths

The validation sample was relatively small with only 95 *BRCA* PVs carriers. Future independent studies should aim to assess the models developed here. The analysis was also restricted to *BRCA1* or *BRCA2*, but gene-panels that include additional susceptibility genes are now widely used, which include additional genes (e.g., *PALB2*) that may be relevant in informing treatment.^3^ However, the present sample size is too small to allow the prediction of carrying PVs in other genes. Although grade was identified as a potential variable to include in risk prediction models, it is noteworthy that quality assurance may be required for this and other variables in order to ensure model accuracy. Finally, whilst ARiCa was shown to perform equally well across different ethnic groups in Malaysia and Singapore, studies in other Asian populations are needed to evaluate its utility in these populations.

Despite the limitations, this is the first study to develop a logistic regression BRCA carrier prediction model and customise clinical genetic testing criteria for use in mainstream germline *BRCA* genetic testing based on unselected sample of breast cancer patients in South East Asia.

## Conclusion

With the advent of germline genetic testing for treatment selection, more women may consider genetic testing as part of their treatment plans. Given that Asian women have a younger age of diagnosis for breast cancer and different distribution of breast cancer subtypes compared to women of European descent, population-specific customisation of BRCA carrier prediction tools is important to enable more accurate BRCA mutation prediction in diverse populations.

## Supplementary Material

Supplemental

## Figures and Tables

**Table 1:** Multivariable regression of ARiCa

Variable	Category	*BRCA* vs Non-*BRCA* n=5,714)				*BRCA1* vs *Non-BRCA1* (n=5,714)				*BRCA2* vs *Non-BRCA2* (n=5,714)			
		Odds Ratio	95% CI		P- value	Odds Ratio	95% CI		P- value	Odds Ratio	95% CI		P- value
Age*		**0.94**	**0.93**	**0.96**	**<0.001**	**0.93**	**0.91**	**0.95**	**<0.001**	**0.95**	**0.94**	**0.97**	**<0.001**
**Ethnicity**	Chinese	1.00	-	-	-	1.00	-	-	-	1.00	-	-	-
	**Malay**	1.26	0.87	1.82	0.220	**1.86**	**1.05**	**3.29**	**0.034**	0.98	0.61	1.57	0.920
	**Indian**	**2.06**	**1.37**	**3.09**	**<0.001**	**3.20**	**1.75**	**5.82**	**<0.001**	1.35	0.77	2.36	0.295
	Other	1.50	0.45	4.96	0.511	1.79	0.23	13.91	0.58	1.37	0.33	5.77	0.667
**Bilateral**	Unilateral	1.00	-	-	-	1.00	-	-	-	1.00	-	-	-
	**Contralateral**	**2.56**	**1.57**	**4.17**	**<0.001**	**4.31**	**2.15**	**8.61**	**<0.001**	1.56	0.80	3.06	0.195
	Ipsilateral	1.21	0.46	3.21	0.689	1.70	0.45	6.49	0.437	0.86	0.20	3.59	0.833
**ER**	Er+	1.00	-	-	-	1.00	-	-	-	1.00	-	-	-
	**ER-**	**1.62**	**1.17**	**2.24**	**0.004**	**5.59**	**3.15**	**9.92**	**<0.001**	0.71	0.45	1.10	0.126
**HER2**	HER2+	1.00	-	-	-	1.00	-	-	-	1.00	-	-	-
	**HER2-**	**2.35**	**1.59**	**3.48**	**<0.001**	**3.11**	**1.61**	**6.01**	**0.001**	**1.84**	**1.13**	**3.00**	**0.015**
**Grade**	One	1.00	-	-	-	1.00	-	-	-	1.00	-	-	-
	**Two**	**3.18**	**1.38**	**7.32**	**0.006**	2.27	0.41	12.49	0.346	**3.62**	**1.39**	**9.39**	**0.008**
	**Three**	**4.02**	**1.72**	**9.42**	**0.001**	2.91	0.53	16.02	0.219	**4.34**	**1.62**	**11.66**	**0.004**
**FHBC**	No	1.00	-	-	-	1.00	-	-	-	1.00	-	-	-
	**Yes**	**3.01**	**2.23**	**4.07**	**<0.001**	**3.48**	**2.13**	**5.69**	**<0.001**	**2.56**	**1.77**	**3.71**	**<0.001**
**FHOC**	No	1.00	-	-	-	1.00	-	-	-	1.00	-	-	-
	**Yes**	**4.57**	**2.51**	**8.30**	**<0.001**	**7.95**	**3.63**	**17.41**	**<0.001**	1.93	0.75	4.92	0.170

Sample: 5714 MyBrCa and SGBCC breast cancer patients in multiply imputed training set.

Abbreviations: Bilateral, Bilateral Breast Cancer; FHBC, First Degree Family History for Breast Cancer; FHOC, First Degree Family History for Ovarian Cancer; 95% CI, 95% Confidence Interval.

* Age of diagnosis for breast cancer of proband.

**Table 2:** Performance of BRCA carrier prediction models at different thresholds

Threshold (%)	Model	*BRCA* vs Non-*BRCA* (n=2,426)		*BRCA1* vs *Non-BRCA1* (n=2,426)		*BRCA2* vs *Non-BRCA2* (n=2,426)	
		Sensitivity (%)	Specificity (%)	Sensitivity (%)	Specificity (%)	Sensitivity (%)	Specificity (%)
**4.0** *	**ARiCa**	71	71	83	70	66	70
**8.0** *	**PENNII**	63	70	83	70	51	69
**2.2** *	**BOADICEA**	66	67	83	67	56	67
**4.0** *	**KOHCal**	66	68	83	67	56	67
**10.0** **	**ARiCa**	34	94	58	93	19	93
	**PENNII**	60	74	78	73	49	73
	**BOADICEA**	22	95	44	95	8	95
	**KOHCal**	44	88	64	87	32	87
**20.0** **	**ARiCa**	15	99	25	98	8	98
	**PENNII**	15	99	25	98	8	98
	**BOADICEA**	7	99	14	99	3	99
	**KOHCal**	22	97	42	97	10	96

Sample: 2426 MyBrCa and SGBCC breast cancer patients with pedigree available in single imputed validation set.

* Optimal threshold.

** Conventional threshold.

**Table 3:** Evaluation of clinical criteria with grade

Criteria	BC + Grade*	TNBC*	Bilateral*	OC	FHBC*	FHOC*	Eligible patients (%)**	*BRCA* PVs carriers (%)**	Detection ratio
**MCC 29**	≤40	≤45	≤60				30.0	63.0	12 : 1
**MCC 17**	≤40	≤60	≤60		≤60	≤60	31.0	64.0	13 : 1
**MCC 33**	≤40	≤50	≤60				32.0	66.0	12 : 1
**NCCN with grade**	≤45	≤60	46-50		46-50		33.0	69.0	12 : 1
**MCC 45**	≤40		≤60				38.0	71.0	14 : 1
**MCC 10**	≤45	≤50	≤60		≤60	≤60	36.0	71.0	13 : 1

Sample: 2448 MyBrCa and SGBCC breast cancer patients in single imputed validation set.

Abbreviations: MCC, Modified Clinical Criteria; BC, Breast Cancer of proband; TNBC, Triple Negative Breast Cancer; Bilateral, Bilateral Breast Cancer; OC, Ovarian Cancer; FHBC, one or more first degree relatives with Breast Cancer; FHOC, one or more first degree relatives with Ovarian Cancer.

* Age of diagnosis for breast cancer of proband.

** Fulfilled at least one criterion.

## Context Summary

### Key objective

Increasing breast cancer incidence and limited resources pose a significant challenge to genetic counselling and testing in many low- and middle-income countries in Asia. Whilst existing mutation prediction models underestimate proportion of carriers in Asian women, a logistic regression model was developed and validated based on South East Asian breast cancer patients unselected for age of diagnosis and family history of cancer to estimate the likelihood of carrying a pathogenic variant in *BRCA1* or *BRCA2* gene, called ARiCa (**A**sian **Ri**sk **Ca**lculator).

### Knowledge generated

ARiCa outperformed existing mutation prediction models. Discrimination of mutation prediction model and efficacy of clinical genetic testing criteria were significantly enhanced by the inclusion of tumour grade.

### Relevance

Population-specific customisation of mutation prediction tools is important to enable more accurate BRCA mutation prediction in diverse populations for referral of breast cancer patients for genetic counselling and testing who may benefit from the selection of therapy.
