# Supplementary material for: Predicting the likelihood of carrying a BRCA1 or BRCA2 mutation in Asian breast cancer patients
Source: J Clin Oncol. Author manuscript; Available in PMC 2023 Mar 2. (PMC7614269; doi:10.1200/JCO.21.01647)
Supplement: Supplemental [file EMS166754-supplement-Supplemental.pdf]

SUPPLEMENTAL METHODS

Study population

Participants donated a blood or saliva sample that was processed and stored, completed a questionnaire that included information on lifestyle risk factors for breast cancer, and provided written informed consent. Germline DNA were sequenced in two batches, using targeted sequencing panels that target the coding regions and exon-intron boundaries of known and suspected breast cancer susceptibility gene, which included *BRCA1* and *BRCA2* (*BRCA*) genes.[1] Target enrichment was performed using the Fluidgm Access Array system (n=2,441) or the Fluidgm Juno system (n=5,721) and sequenced on Illumina HiSeq 2500 or HiSeq 4000. All identified protein truncating variants and Class 4 and Class 5 missense variants were confirmed by Sanger sequencing and were considered as pathogenic variant (PV) for the purpose of this study. Recruitment and genetic studies were approved by the Ethics Committees of University Malaya Medical Centre [UM 842.9], Subang Jaya Medical Centre [reference no:201208.1], NHG Domain Specific Review Board [NHG DSRB Ref:2009/00501], and SingHealth Centralised Institutional Review Board [CIRB Ref:2010/632/B].

Statistical analyses

Existing BRCA carrier prediction models

The predicted likelihood of carrying a *BRCA* PV was generated for each patient by using batch version for BOADICEA 5.0 which is implemented in the CanRisk tool,[2] including personal (cancer history, demographic, pathology, lifestyle, anthropometric, menstrual and reproductive) and family history information (cancer history and demographic) based

on UK incidences,[3] using web application for PENNII,[4] or based on beta-coefficients from multivariable logistic regression for KOHCal.[5]

Development and validation of population-specific BRCA carrier prediction model

Independent variables that were considered for the Asian Risk Calculator model development, include age of diagnosis for breast cancer, ethnicity (Chinese/Malay/Indian/Other), bilateral breast cancer, pathological features, grade, immune-histochemical subtypes, and presence of first-degree family history of breast cancer or ovarian cancer. Pathological features include estrogen receptor (ER), progesterone receptor (PR), and human epidermal growth factor receptor 2 (HER2) evaluated separately or in combination. Grade index of carcinomas assigned based on the morphology of cancer cells from excised tumours viewed under microscope were obtained from histopathological reports: grade 1 for well-differentiated carcinomas, grade 2 for moderately differentiated carcinomas, and grade 3 for poorly differentiated carcinomas. The training and validation sets were selected randomly but such that the prevalence of *BRCA* PV was the same (4%) in both sets. Equality tests were performed to ensure that the distributions of independent and outcome variables were similar between training and validation sets.

Tumour biomarkers and grade had missing rates greater than 10%: 11% for ER, 14% for PR, 26% for HER2, and 15% for grade **(Supplementary Table 1).** First-degree family history of ovarian cancer, parity, menopausal status, and BMI were also included in the imputation model as they were shown to be important predictors of the independent

variables in this study. We evaluated the MAR assumption by assessing the association of missingness status of each variable by remaining variables included in the imputation model (data not shown). We generated 100 imputed datasets for analyses. Hormone receptor (HR+:ER+, PR+), TNBC, and immune-histochemical subtypes were not directly imputed, but instead derived from the imputed ER, PR, and HER2 status data. Classification of immune-histochemical subtypes are as follow: TNBC (ER-, PR-, HER2-), HER2-enriched (ER-, PR-, HER2+), Luminal A (ER+/-, PR+/-, HER2-), and Luminal B (ER+/-, PR+/-, HER2+). Each imputed dataset was analysed separately and combined according to Rubin's rules.[6] The proportion of missing data in the validation set was similar to that in the training set **(Supplementary Table 1).**

The area under receiver operating curve (AUC) was used to assess the ability to discriminate *BRCA* PV carriers from noncarriers. AUC values range from 0 to 1, with AUC of 1 indicating perfect discrimination.[7] The Hosmer-Lemeshow (HL) test was performed to measure calibration that provides an indication of the overall fit of models to the data by comparing the observed and expected number of *BRCA* PV carriers in deciles of predicted carrier probabilities.[8] Our analyses considered that pathogenic variants are protein truncating variants and known pathogenic missense variants. Since the BRCA testing did not involve screening for large re-arrangements the mutation testing sensitivity will be somewhat lower than 100%, however large re-arrangements account for a small proportion of *BRCA* PV. Our modelling approach assumed that the mutation testing sensitivity is 100%. Performance measures reported, included sensitivity/detection rate (*BRCA* PV carriers detected, %), specificity (noncarriers detected, %), screening rate

(eligible patients, %), and detection ratio (number of patients to be screened to detect one carrier).

Customisation and evaluation of Modified Clinical Criteria for germline BRCA genetic testing

Combinations of age of diagnosis of proband in years are as follow: a) breast cancer diagnosed at age (≤40, ≤45, ≤50, ≤55) or grade 2 or 3 breast cancer diagnosed at age (≤40, ≤45, ≤50, ≤55), b) TNBC (≤40, ≤45, ≤50, ≤55, ≤60, any age), c) bilateral breast cancer (≤60, any age), d) one or more first-degree relatives with breast cancer (≤60, any age), e) one or more first-degree relatives with ovarian cancer (≤60, any age). Screening rate (eligible patients, %) and sensitivity/detection rate (*BRCA* PV carriers detected, %) were calculated for each criterion as well as for the overall clinical criteria whereby at least one criterion was fulfilled. To enable direct comparison with mutation prediction models, the efficacy of MCC was evaluated in the validation set by comparing detection ratio (number of patients to be screened to detect one carrier).

SUPPLEMENTAL MATERIALS

Supplementary Table 1. Characteristics of study population

Variable	Total n (%) (N=8,162)	Missing** n (%)	Chinese n (%) (n=6,140)	Malay n (%) (n=1,207)	Indian n (%) (n=718)	*P*
**Age***	52.26 (10.77)	56 (0.7)	52.79 (10.80)	49.28 (10.19)	52.53 (10.53)	**0.031**
**Ethnicity**	-	14 (0.2)	-	-	-	-
Chinese	6140 (75.4)	-	-	-	-	-
Malay	1207 (14.8)	-	-	-	-	-
Indian	718 (8.8)	-	-	-	-	-
Other	83 (1.0)	-	-	-	-	-
**Bilateral**	-	55 (0.7)	-	-	-	0.051
Unilateral	7621 (94.0)	-	5727 (94.0)	1138 (94.5)	671 (93.6)	-
Contralateral	352 (4.3)	-	252 (4.1)	55 (4.6)	38 (5.3)	-
Ipsilateral	134 (1.7)	-	115 (1.9)	11 (0.9)	8 (1.1)	-
**ER**	-	917 (11.2)	-	-	-	**<0.001**
ER+	5171 (71.4)	-	3945 (72.8)	743 (67.8)	426 (66.6)	-
ER-	2074 (28.6)	-	1477 (27.2)	355 (32.2)	214 (33.4)	-
**PR**	-	1182 (14.5)	-	-	-	**0.029**
PR+	4394 (62.9)	-	3344 (63.9)	635 (59.8)	375 (61.6)	-
PR-	2586 (37.1)	-	1885 (36.1)	426 (40.2)	234 (38.4)	-
**HR**	-	1186 (14.5)	-	-	-	**0.003**
HR+	5215 (74.8)	-	3962 (75.8)	762 (72.0)	434 (71.3)	-
HR-	1761 (25.2)	-	1264 (24.2)	298 (28.0)	175 (28.7)	-
**HER2**	-	2141 (26.2)	-	-	-	**0.001**
HER2+	1821 (30.3)	-	1330 (29.7)	327 (35.0)	147 (27.3)	-
HER2-	4200 (69.7)	-	3149 (70.3)	605 (65.0)	391 (72.7)	-
**Grade**	-	1221 (15.0)	-	-	-	**0.002**
One	986 (14.2)	-	779 (14.8)	116 (11.4)	85 (14.4)	-
Two	3082 (44.4)	-	2363 (44.9)	429 (42.1)	256 (43.2)	-
Three	2874 (41.4)	-	2116 (40.3)	474 (46.5)	251 (42.4)	-
**Subtypes**	-	2340 (28.7)	-	-	-	**<0.001**
Luminal A	3316 (57.0)	-	2527 (58.2)	460 (51.1)	289 (56.3)	-
Luminal B	975 (16.7)	-	716 (16.5)	181 (20.1)	70 (13.6)	-
TNBC	798 (13.7)	-	560 (12.9)	135 (15.0)	88 (17.2)	-
HER2-enriched	733 (12.6)	-	535 (12.4)	124 (13.8)	66 (12.9)	-
**TNBC**	-	1148 (14.1)	-	-	-	**0.007**
No	6216 (88.6)	-	4695 (89.3)	925 (87.3)	527 (85.7)	-
Yes	798 (11.4)	-	560 (10.7)	135 (12.7)	88 (14.3)	-
**FHBC**	-	117 (1.4)	-	-	-	**0.008**
No	6823 (84.8)	-	5085 (84.2)	1046 (87.6)	611 (85.8)	-
Yes	1222 (15.2)	-	957 (15.8)	148 (12.4)	101 (14.2)	-
**FHOC**	-	-	-	-	-	0.203
No	8031 (98.4)	-	6042 (98.4)	1191 (98.7)	701 (97.6)	-
Yes	131 (1.6)	-	98 (1.6)	16 (1.3)	17 (2.4)	-
**Carrier**	-	-	-	-	-	**<0.001**
Noncarrier	7839 (96.0)	-	5.942 (96.8)	1136 (94.1)	668 (93.0)	-
BRCA1	122 (1.5)	-	66 (1.1)	29 (2.4)	25 (3.5)	-
BRCA2	201 (2.5)	-	132 (2.1)	42 (3.5)	25 (3.5)	-

NOTE. Sample: 8,162 Malaysian Breast Cancer Genetic Study and Singapore Breast Cancer Cohort breast cancer patients before imputation.

Abbreviations: Bilateral, bilateral breast cancer; Carrier, BRCA pathogenic variant carrier status; ER, estrogen receptor; FHBC, first-degree family history for breast cancer; FHOC, first-degree family history for ovarian cancer; HER2, human epidermal growth factor receptor 2; HR, hormone receptor; PR, progesterone receptor; Subtypes, immune-histochemical subtypes; TNBC, triple-negative breast cancer.

*Age of diagnosis for breast cancer of proband in mean (standard deviation).

***Training set: age of diagnosis (0.8%), ethnicity (0.1%), bilateral breast cancer (0.6%), ER (11%), PR (14%), HER2 (26%), grade (15%), and first-degree family history of breast cancer (1%).

***Validation set: age of diagnosis (0.5%), ethnicity (0.3%), bilateral breast cancer (0.7%), ER (11%), PR (15%), HER2 (26%), grade (16%), and first-degree family history of breast cancer (2%).

Supplementary Table 2. Comparison of *BRCA* pathogenic variant carrier status

Variable	*BRCA1* n (%) (n=122)	Non-*BRCA1* n (%) (n=8,040)	*P*	*BRCA2* n (%) (n=201)	Non-*BRCA2* n (%) (n=7,961)	*P*
**Age***	43.93 (10.78)	52.39 (10.72)	**<0.001**	47.59 (10.16)	52.38 (10.75)	**<0.001**
**Ethnicity**	-	-	**<0.001**	-	-	**0.013**
Chinese	66 (54.1)	6074 (75.7)	-	132 (65.7)	6008 (75.6)	-
Malay	29 (23.8)	1178 (14.7)	-	42 (20.9)	1165 (14.7)	-
Indian	25 (20.5)	693 (8.6)	-	25 (12.4)	693 (8.7)	-
Other	2 (1.6)	81 (1.0)	-	2 (1.0)	81 (1.0)	-
**Bilateral**	-	-	**<0.001**	-	-	**0.035**
Unilateral	100 (82.0)	7521 (94.2)	-	180 (90.5)	7441 (94.1)	-
Contralateral	18 (14.8)	334 (4.2)	-	16 (8.0)	336 (4.2)	-
Ipsilateral	4 (3.2)	130 (1.6)	-	3 (1.5)	131 (1.7)	-
**ER**	-	-	**<0.001**	-	-	0.340
ER+	25 (23.4)	5146 (72.1)	-	132 (74.6)	5039 (71.3)	-
ER-	82 (76.6)	1992 (27.9)	-	45 (25.4)	2029 (28.7)	-
**PR**	-	-	**<0.001**	-	-	0.902
PR+	23 (22.1)	4371 (63.6)	-	105 (62.5)	4289 (63.0)	-
PR-	81 (77.9)	2505 (36.4)	-	63 (37.5)	2523 (37.0)	-
**HR**	-	-	**<0.001**	-	-	0.249
HR+	30 (28.8)	5185 (75.5)	-	132 (78.6)	5083 (74.7)	-
HR-	74 (71.2)	1687 (24.5)	-	35 (21.4)	1725 (25.3)	-
**HER2**	-	-	**<0.001**	-	-	**<0.001**
HER2+	13 (13.3)	1808 (30.5)	-	25 (17.1)	1796 (30.6)	-
HER2-	85 (86.7)	4115 (69.5)	-	121 (82.9)	4079 (69.4)	-
**Grade**	-	-	**<0.001**	-	-	**<0.001**
One	2 (2.0)	984 (14.4)	-	4 (2.4)	982 (14.5)	-
Two	27 (27.6)	3054 (44.6)	-	83 (49.7)	2998 (44.3)	-
Three	69 (70.4)	2805 (39.0)	-	80 (47.9)	2794 (41.2)	-
**Subtypes**	-	-	**<0.001**	-	-	**0.001**
Luminal A	20 (21.5)	3296 (57.5)	-	92 (65.7)	3224 (56.7)	-
Luminal B	6 (6.5)	969 (17.0)	-	17 (12.1)	958 (16.9)	-
TNBC	63 (67.7)	735 (12.8)	-	26 (18.6)	772 (13.6)	-
HER2-enriched	4 (4.3)	729 (12.7)	-	5 (3.6)	728 (12.8)	-
**TNBC**	-	-	**<0.001**	-	-	0.118
No	38 (37.6)	6178 (89.4)	-	146 (84.9)	6070 (88.7)	-
Yes	63 (62.4)	735 (10.6)	-	26 (15.1)	772 (11.3)	-
**FHBC**	-	-	**<0.001**	-	-	**<0.001**
No	75 (62.0)	6748 (85.2)	-	141 (70.5)	6682 (85.2)	-
Yes	46 (38.0)	1176 (14.8)	-	59 (29.5)	1163 (14.8)	-
**FHOC**	-	-	**<0.001**	-	-	**0.001**
No	106 (86.9)	7925 (98.6)	-	192 (95.5)	7839 (98.5)	-
Yes	16 (13.1)	115 (1.4)	-	9 (4.5)	122 (1.5)	-

NOTE. Sample: 8,162 Malaysian Breast Cancer Genetic Study and Singapore Breast Cancer Cohort breast cancer patients before imputation.
Abbreviations: Bilateral, bilateral breast cancer; ER, estrogen receptor; FHBC, first-degree family history for breast cancer; FHOC, first-degree family history for ovarian cancer; HER2, human epidermal growth factor receptor 2; HR, hormone receptor; PR, progesterone receptor; Subtypes, immune-histochemical subtypes; TNBC, triple-negative breast cancer.
*Age of diagnosis for breast cancer of proband in mean (standard deviation).

Supplementary Fig 1. Study design and sample selection

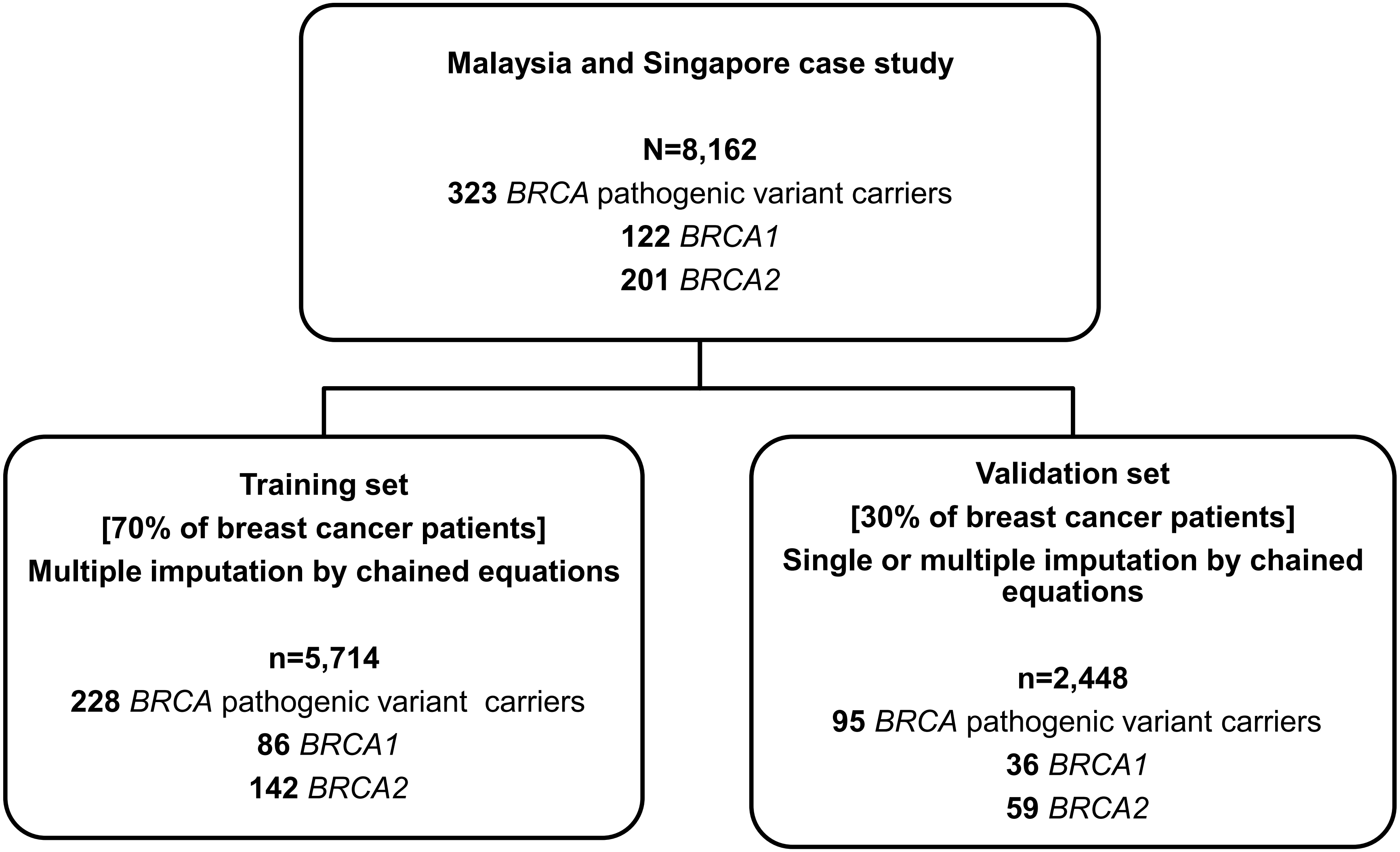

Supplementary Table 3. Discrimination and calibration of multivariable regression models for selection of Asian Risk Calculator

Model	***BRCA*** **Versus Non-*BRCA*** **(*n*=2,448)**		***BRCA1*** **Versus Non-*BRCA1*** **(*n*=2,448)**		***BRCA2*** **Versus Non-*BRCA2*** **(*n*=2,448)**	
	**AUC (95% CI)**	**HL (*P*)**	**AUC (95% CI)**	**HL (*P*)**	**AUC (95% CI)**	**HL (*P*)**
**Single imputation**						
**(1) TNBC**	0.78 (0.74 - 0.83)	7.79 (0.454)	**0.86 (0.79 - 0.93)**	**2.63 (0.955)**	0.72 (0.66 - 0.77)	6.19 (0.626)
**(2) ER**	0.77 (0.72 - 0.82)	2.56 (0.959)	0.84 (0.77 - 0.91)	5.28 (0.728)	0.72 (0.66 - 0.78)	12.57 (0.128)
**(3) ER + HER2**	**0.80 (0.75 - 0.84)**	**5.43 (0.711)**	**0.86 (0.79 - 0.93)**	**4.33 (0.826)**	**0.75 (0.69 - 0.80)**	**12.15 (0.145)**
**(4) HR + HER2**	0.79 (0.75 - 0.84)	4.22 (0.836)	0.86 (0.79 - 0.93)	6.12 (0.634)	0.74 (0.69 - 0.80)	12.26 (0.140)
**(5) HER2**	0.79 (0.75 - 0.84)	6.85 (0.552)	0.85 (0.79 - 0.90)	12.45 (0.132)	**0.75 (0.69 - 0.80)**	**10.89 (0.208)**
**(6) Subtypes**	0.80 (0.75 - 0.84)	7.81 (0.452)	0.86 (0.79 - 0.92)	3.82 (0.873)	0.75 (0.69 - 0.80)	15.14 (0.056)
**Multiple imputation**						
**(1) TNBC**	0.78 (0.77 - 0.78)	6.36 (0.701)	0.85 (0.84 - 0.85)	5.39 (0.878)	0.71 (0.70 - 0.71)	5.25 (0.786)
**(2) ER**	0.77 (0.76 - 0.77)	3.73 (0.926)	0.84 (0.83 - 0.84)	5.86 (0.805)	0.71 (0.70 - 0.71)	13.75 (0.185)
**(3) ER + HER2**	**0.80 (0.79 - 0.80)**	**5.08 (0.937)**	**0.86 (0.85 - 0.86)**	**5.56 (0.824)**	**0.73 (0.72 - 0.73)**	**8.79 (0.689)**
**(4) HR + HER2**	0.78 (0.77 - 0.78)	5.12 (0.946)	0.85 (0.84 - 0.85)	5.25 (0.887)	0.72 (0.71 - 0.72)	7.02 (0.831)
**(5) HER2**	0.78 (0.77 - 0.78)	6.78 (0.842)	0.84 (0.83 - 0.84)	7.20 (0.938)	0.72 (0.71 - 0.72)	9.01 (0.485)
**(6) Subtypes**	0.79 (0.78 - 0.79)	5.90 (0.756)	0.85 (0.84 - 0.85)	4.75 (0.816)	0.72 (0.71 - 0.72)	10.42 (0.563)

NOTE. Sample: 2,448 Malaysian Breast Cancer Genetic Study and Singapore Breast Cancer Cohort breast cancer patients in imputed validation set.
Abbreviations: AUC, area under receiver operating curve; ER, estrogen receptor; HER2, human epidermal growth factor receptor 2; HL, Hosmer-Lemeshow; HR, hormone receptor; Subtypes, immune-histochemical subtypes; TNBC, triple-negative breast cancer.

Supplementary Table 4. Discrimination and calibration of Asian Risk Calculator by ethnicity

Mutation	Chinese (*n*=1,816)		Malay (*n*=361)		Indian (*n*=248)	
	**AUC (95% CI)**	**HL (*P*)**	**AUC (95% CI)**	**HL (*P*)**	**AUC (95% CI)**	**HL (*P*)**
**Single imputation**						
BRCA* Versus Non-*BRCA	0.80 (0.74 - 0.86)	9.57 (0.297)	0.73 (0.63 - 0.83)	6.09 (0.637)	0.77 (0.65 - 0.88)	6.01 (0.647)
BRCA1* Versus Non-*BRCA1	0.88 (0.80 - 0.96)	6.10 (0.636)	0.73 (0.51 - 0.95)	9.37 (0.312)	0.91 (0.81 - 1.00)	1.78 (0.987)
BRCA2* Versus Non-*BRCA2	0.76 (0.69 - 0.84)	6.14 (0.631)	0.72 (0.60 - 0.83)	5.86 (0.663)	0.69 (0.57 - 0.80)	9.85 (0.276)
**Multiple imputation**						
BRCA* Versus Non-*BRCA	0.79 (0.77 - 0.81)	7.97 (0.635)	0.75 (0.71 - 0.79)	5.92 (0.945)	0.77 (0.75 - 0.79)	5.25 (0.821)
BRCA1* Versus Non-*BRCA1	0.86 (0.84 - 0.88)	5.41 (0.826)	0.79 (0.73 - 0.85)	8.60 (0.712)	0.91 (0.90 - 0.92)	1.82 (0.987)
BRCA2* Versus Non-*BRCA2	0.76 (0.74 - 0.78)	7.17 (0.785)	0.68 (0.64 - 0.72)	6.28 (0.909)	0.70 (0.66 - 0.74)	8.64 (0.655)

NOTE. Sample: 2,448 Malaysian Breast Cancer Genetic Study and Singapore Breast Cancer Cohort breast cancer patients in imputed validation set.
Abbreviations: AUC, area under receiver operating curve; HL, Hosmer-Lemeshow.

Supplementary Fig 2. Optimal carrier probability threshold of Asian Risk Calculator

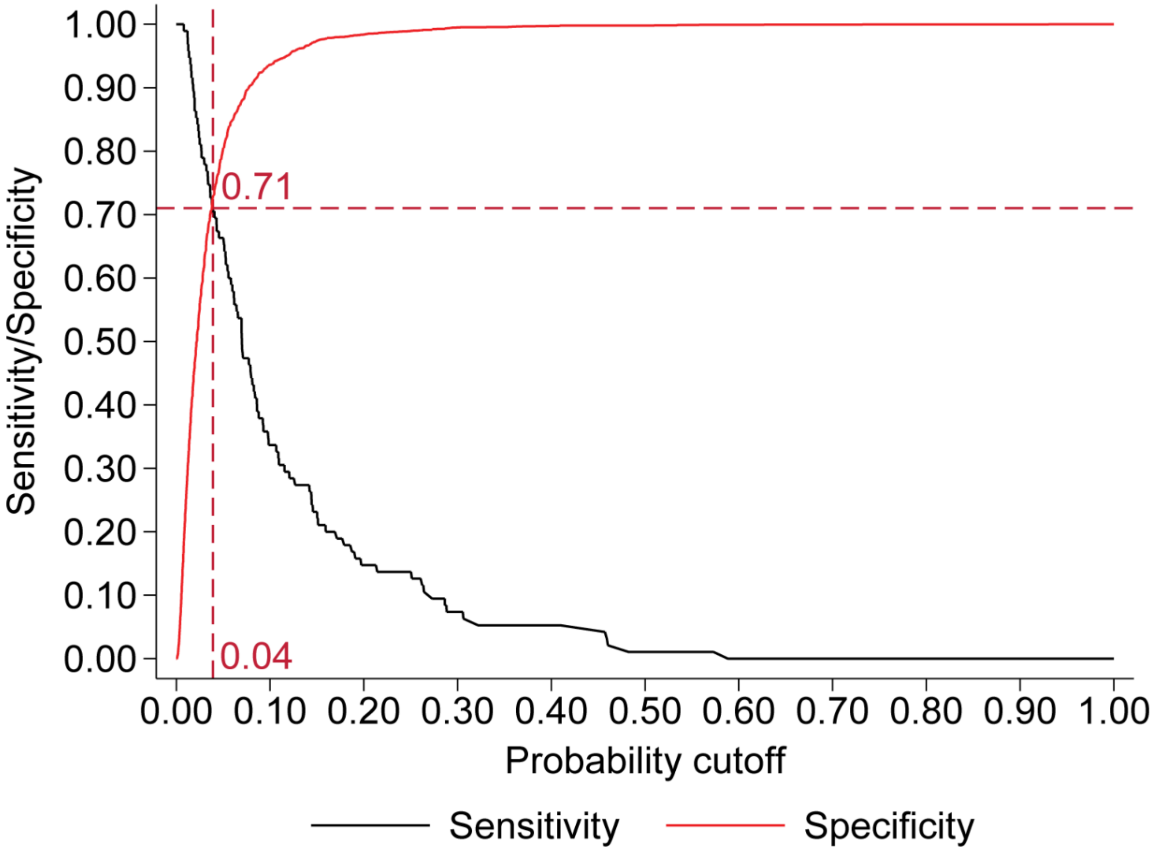

NOTE. Sample: 2,448 Malaysian Breast Cancer Genetic Study and Singapore Breast Cancer Cohort breast cancer patients in single imputed validation set.

Supplementary Table 5. Performance of Asian Risk Calculator at optimal carrier probability threshold

Model		Single imputation (n=2,448)	Multiple imputation (n=2,448)
**Threshold (%)**		4.0	4.0
**Sensitivity (%)**			
	Overall *BRCA*	71 (61 - 80)*	70 (69 - 70)**
	BRCA1	83 (67 - 94)*	84 (83 - 85)**
	BRCA2	66 (53 - 78)*	63 (62 - 64)**
**Specificity (%)**			
	Overall *BRCA*	71 (69 - 73)*	71 (70 - 71)**
	BRCA1	70 (68 - 72)*	69 (68 - 69)**
	BRCA2	70 (68 - 72)*	70 (69 - 70)**
**Eligible patients (%)**		31 (29 - 33)*	31 (30 - 31)**
**Detection ratio**		11 : 1	11 : 1

NOTE. Sample: 2,448 Malaysian Breast Cancer Genetic Study and Singapore Breast Cancer Cohort breast cancer patients in imputed validation set.
*95% confidence intervals generated using normal approximation to binomial distribution with continuity correction.
**95% confidence intervals generated using average of 100 imputed validation sets.

Supplementary Fig 3. Observed proportion and expected probability of BRCA carrier prediction models by type of germline *BRCA* pathogenic variant

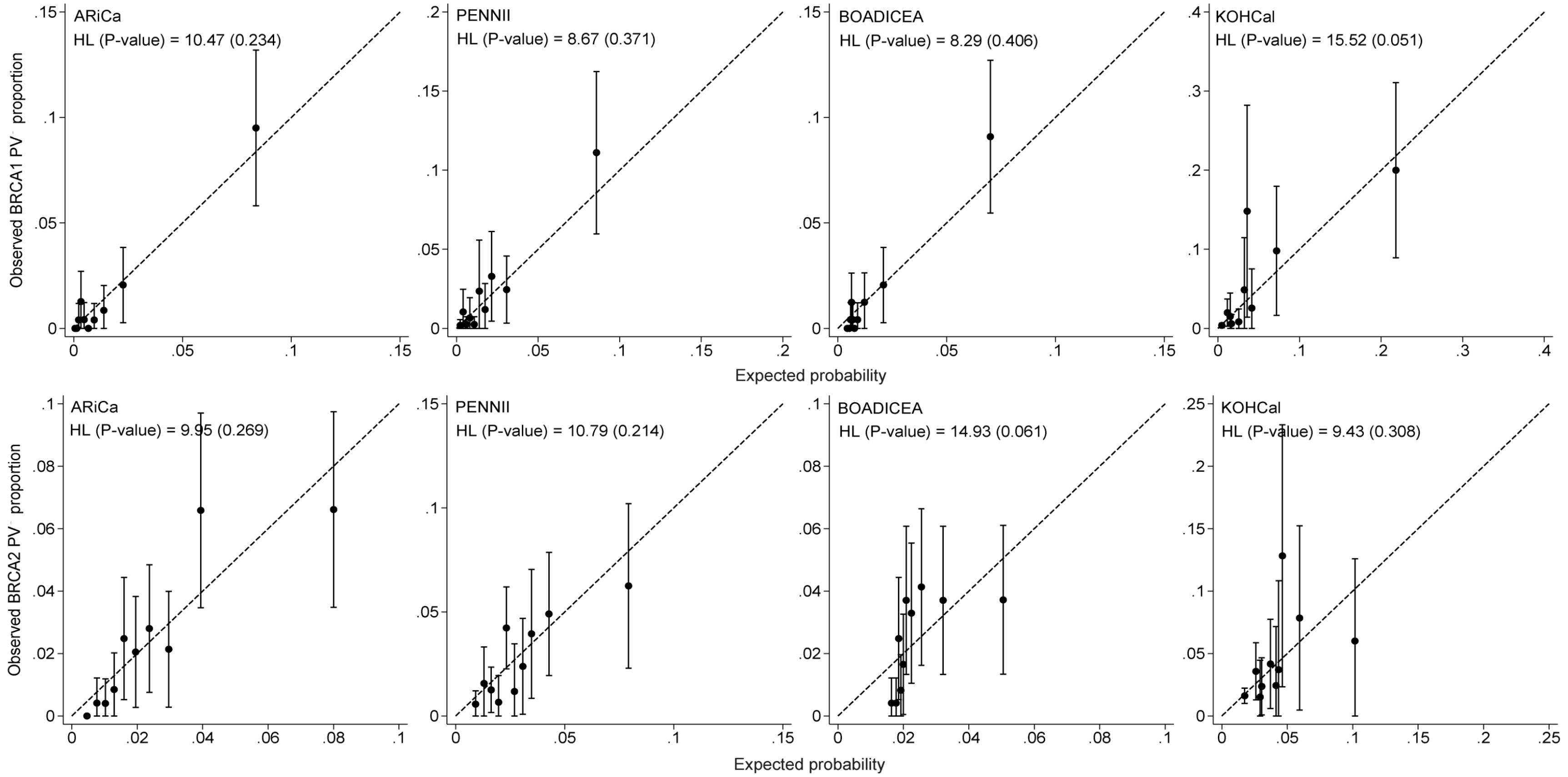

NOTE. Sample: 2,426 Malaysian Breast Cancer Genetic Study and Singapore Breast Cancer Cohort with pedigree available in single imputed validation set.
Abbreviation: ARiCa, Asian Risk Calculator; HL, Hosmer-Lemeshow; PV, pathogenic variant.

REFERENCES

1. Dorling et al: Breast cancer risk genes: association analysis of rare coding variants in 34 genes in 60,466 cases and 53,461 controls. N Engl J Med, 2021
2. Carver T, Hartley S, Lee A, et al: CanRisk Tool—A Web Interface for the Prediction of Breast and Ovarian Cancer Risk and the Likelihood of Carrying Genetic Pathogenic Variants. Cancer Epidemiol Biomarkers Prev 30:469-73, 2021
3. Lee A, Mavaddat N, Wilcox AN, et al: BOADICEA: a comprehensive breast cancer risk prediction model incorporating genetic and nongenetic risk factors. Genet Med 21:1708-1718, 2019
4. Lindor NM, Johnson KJ, Harve H, et al: Predicting BRCA1 and BRCA2 gene mutation carriers: comparison of PENN II model to previous study. Fam Cancer 9:495-502, 2010
5. Kang E, Park SK, Lee, JW, et al: KOHBRA BRCA risk calculator (KOHCal): a model for predicting BRCA1 and BRCA2 mutations in Korean breast cancer patients. J Hum Genet, 61:365-371, 2016
6. Little RJ, Rubin DB: Statistical analysis with missing data (Vol. 793). John Wiley & Sons, 2019
7. Hanley JA, McNeil BJ: The meaning and use of the area under a receiver operating characteristic (ROC) curve. Radiology 143:29-36, 1982
8. Archer KJ, Lemeshow S: Goodness-of-fit test for a logistic regression model fitted using survey sample data. Stata J 6:97-105, 2006
